# Association between MTHFR C677T and A1298C Polymorphisms and NSCL/P Risk in Asians: A Meta-Analysis

**DOI:** 10.1371/journal.pone.0088242

**Published:** 2014-03-21

**Authors:** Mengmeng Zhao, Yangwu Ren, Li Shen, Yue Zhang, Baosen Zhou

**Affiliations:** 1 Department of Epidemiology, School of Public Health, China Medical University, Shenyang, Liaoning Province, China; 2 Key Laboratory of Cancer Etiology and Intervention, University of Liaoning Province, Shenyang, China; Nanjing Medical University, China

## Abstract

**Objective:**

Several studies have reported the association between methylenetetrahydrofolate reductase (MTHFR) C677T and A1298C polymorphisms and nonsyndromic cleft lip with or without palate (NSCL/P) in Asian populations. However, findings have been conflicting. In order to investigate the association, a meta-analysis was performed.

**Methods:**

We searched Pubmed, MedLine and EmBase database to selected eligible studies. The pooled odds ratios (ORs) with 95% confidence intervals (95%CIs) were calculated using fixed effects model or random effects model to assess the association between MTHFR polymorphisms and NSCL/P in both Asian children and mothers.

**Results:**

Finally, nine case-control studies were included. Overall, the MTHFR C677T polymorphism and NSCL/P showed pooled ORs (95%CI) of 1.41(1.23–1.61) in Asian children, and 1.70(1.19–2.42) in Asian mothers. Subgroup analyses by geographical locations further identified the association in Eastern Asian children, Western/Central Asian children and mothers, but not in Eastern Asian mothers. However, no significant relationship between MTHFR A1298C polymorphism and NSCL/P was found in this meta-analysis.

**Conclusions:**

The MTHFR 677T allele was associated with an increased risk of NSCL/P in Asian populations.

## Introduction

Nonsymdromic cleft lip with or without palate (NSCL/P) is one of the most common congenital malformations of the head and neck area in the world, occurring in approximately 1 in every 700 live births [1]. Around the globe, the distribution of prevalence rate of NSCL/P is not homogeneous, and higher values are found in parts of Asia (China, Japan) and Latin America, whereas Israel, South Africa and Southern Europe showed the lowest values [2]. The etiology of NSCL/P is considered to be multifactorial, causing by both hereditary factors and environmental factors. Epidemiologic studies revealed that mothers who used multivitamins containing folic acid showed a lower risk of having an offspring with NSCL/P compared with mothers did not use multivitamins [3]. However, it is still unknown which ingredients in multivitamins contribute to this risk reduction. It is widely accepted that folic acid is a key factor in the development of craniofacial structures. Several studies have reported a reduced risk of NSCL/P when mothers used either folic acid supplements or dietary folate during pregnancy [4], [5]. Some other studies, however, have provided variable or even contradictory results [6]–[8]. Therefore, genetic variations in folate metabolism gene were believed to affect individual susceptibility to NSCL/P.

Methylenetetrahydrofolate reductase (MTHFR) is a key enzyme in the metabolism of folate. This enzyme irreversibly catalyzes the reaction of 5, 10-methylenetetrahydrofolate to 5-methyltetrahydrofolate, which is the primary circulating form of folate [6]. The most common single nucleotide polymorphism (SNP) is MTHFR C677T, which results in an alanine to valine exchanges and is associated with reduced enzyme activity. MTHFR A1298C, another common functional polymorphism, also affects enzyme activity [7], [8]. The two SNPs have been studied as candidate genetic factors for NSCL/P risk [9]–[12].

The first report evaluating the role of MTHFR gene polymorphisms in the development of NSCL/P was conducted by Tolarova et al.(1998) [13]. The findings suggested that MTHFR C677T variant genotype increased the risk of NSCL/P. They also found a significant association between MTHFR A1298C polymorphism and NSCL/P. Since then, a great number of studies have been conducted, but the results were inconsistent [14]–[16]. In order to elucidate the role of MTHFR C677T and A1298C polymorphisms in NSCL/P, several meta-analyses were performed [17]–[20], but the relationships especially among Asian subjects remained unclear. Recently, some new case-control studies with large sample size have been published [21]–[23]. Hence, we performed this meta-analysis to derive a more precise estimation of the association of MTHFR C677T and A1298C polymorphisms with NSCL/P in Asian populations.

## Materials and Methods

### Literature search and selection

Eligible literatures were screened from PubMed, MedLine and EmBase database (up to May 31, 2013). We used the following keywords and subject terms: “methylenetetrahydrofolate reductase” or “MTHFR” and “cleft lip” or “cleft palate”. Furthermore, we manually searched references in the eligible articles. There was no language limitation. And the search results were limited to humans.

### Criteria of inclusion and exclusion

Inclusion criteria were showed as following: (1) evaluation the association between MTHFR C677T and/or A1298C polymorphisms and NSCL/P risk in Asian populations; (2) case-control studies; (3) the frequencies of all genotype distribution or other available data for estimating the OR (95% CI). If overlapping cases or controls were presented in multiple studies, the most recent publication or the largest study was included. The exclusion criteria were: (1) not using a case-control study design; (2) had no detailed data on genotype distribution; (3) not in Asian populations.

### Data extraction

The following information was carefully and independently collected from each eligible study by two authors: first author's name, year of publication, country, population of cases and controls, genotyping method, and numbers of each genotype. The difference was settled by reaching an agreement among all authors.

### Statistical analysis

To assess the study quality, Hardy–Weinberg equilibrium (HWE) in controls in each study was calculated by chi-squared test. P value<0.05 was considered a departure from HWE. The associations between MTHFR C677T and A1298C gene polymorphisms and NSCL/P risk were estimated by the odds ratios (ORs), together with the 95% confidence interval (95%CI). The significance of the pooled OR was determined by the Z test, with P<0.05 considered significant. Heterogeneity between studies was assessed by Q test. If P<0.05, the heterogeneity was considered statistically significant. The I^2^ values were used to quantify the percentage of the total variation among studies when heterogeneity was assessed. The I^2^ value ranged from 0 to 100%. 25%, 50%, 75% expressed low, moderate, high heterogeneity, respectively. When I^2^<50%, a fixed effects model was applied to estimate the pooled results. Otherwise, the random-effect model was used.

Publication bias was investigated visually in a funnel plot of log (OR) against its standard error (SE). An asymmetric plot suggested possible publication bias. And the degree of asymmetry was assessed by Egger's test. Sensitivity analysis was performed by omitting each study in turn to assess the results stability.

Software STATA version 11.0 was used for all analyses.

## Results

### Selection of studies

**Figure 1:**
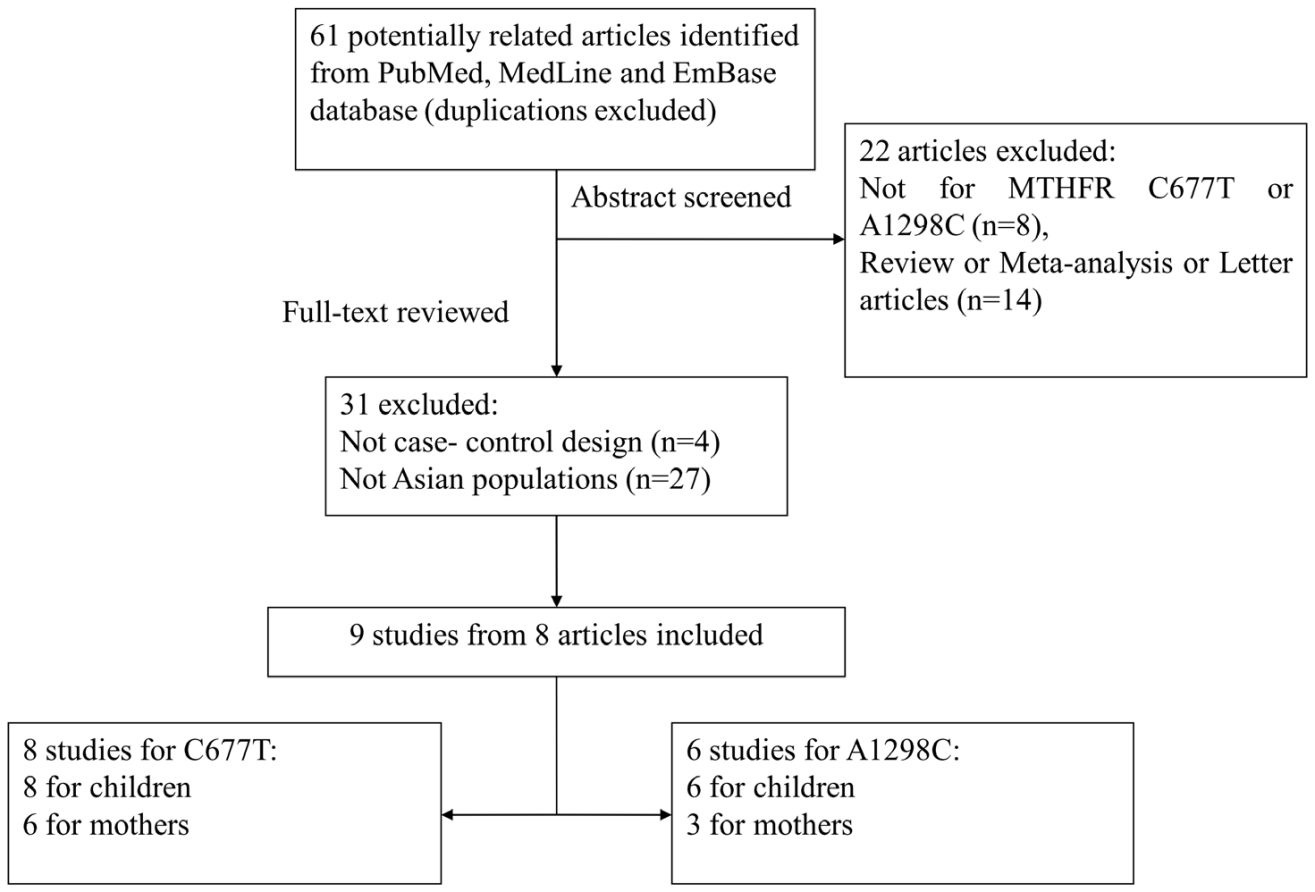
Flow chart explaining the selection of eligible studies included in the meta-analysis.

A flow chart summarizing the process of study selection is shown in Figure 1. Based on the inclusion and exclusion criteria, eight articles were included after full-text reviewing [14], [16], [21]–[26]. Worthy of note, a study by Aida et al.(2012) consisted of two groups of control was treated as two studies [21]. Thus, a total of nine studies from eight articles were included in this meta-analysis. Among them, nine studies provided date on MTHFR C677T polymorphism (eight studies were carried out in Asian children and six in Asian mothers), and 6 studies on A1298C polymorphism (six in Asian children and three in Asian mothers). The main characteristics of selected studies were summarized in Table 1.

**Table 1:** The characteristics of eligible studies included in the meta-analysis.

Anthor(year)	Country	Geographical location	Population of cases	Source of control	Subjects	Sample size		*P* of HWE	
						Case	Control	677	1298
Kumari et al. (2013)	India	Western/Central Asia	Cases having family history of any congenital malformation, kidney-related diseases and other severe diseases were excluded. Mean age was 5 years.	Mixed	Children	467	469	0.518	0.134
Aida et al. (2012)	Turkey	Western/Central Asia	Cases were excluded due to alternative diagnosis of their existing syndromic conditions. Age range not stated.	Mixed	Children	56	76/93^a^	0.105/0.779	0.187
					Mothers	54	76/93		
Wang et al. (2012)	China	Eastern Asia	Not stated.	HB	Mothers	89	64	0.070	
Han et al. (2011)	China	Eastern Asia	Cases with recognized congenital anomalies or syndromes were excluded. Mean age was 9.57±0.72 years.	HB	Children	187	213	0.236	0.545
Guo et al. (2009)	China	Eastern Asia	Cases having family history of any congenital malformation were excluded. Mean age was 9.04 years.	HB	Children	97	104	0.277	
					Mothers	97	104		
Ali et al. (2009)	India	Western/Central Asia	Syndromic patients as well as families with a syndromic member other than NSCL/P probands were not included. Age range for patients 4 months-24 years, for mothers 19–45 years.	PB	Children	323	214	0.916	0.395
					Mothers	116	214		
Wan et al. (2006)	China	Eastern Asia	Cases having family history of any congenital malformation were excluded. Age range not stated.	HB	Children	48	60	0.075	0.210
Shotelersuk et al. (2003)	Thailand	Eastern Asia	All syndromic cases were excluded. Age range not stated.	PB	Children	109	202	0.478	0.876
					Mothers	67	202		

**HWE** Hardy-Weinberg equilibrium; **HB** hospital based; **PB** population based.

a two groups of control.

### Meta analysis

**Figure 2:**
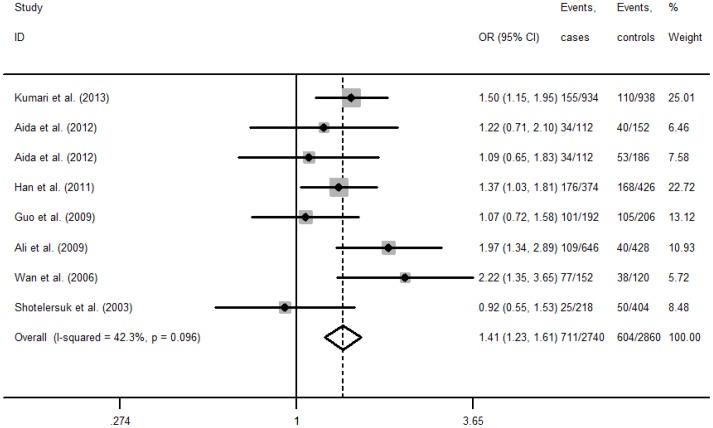
Forest plot of association between MTHFR C677T polymorphism and NSCL/P risk in children (T vs. C).

**Figure 3:**
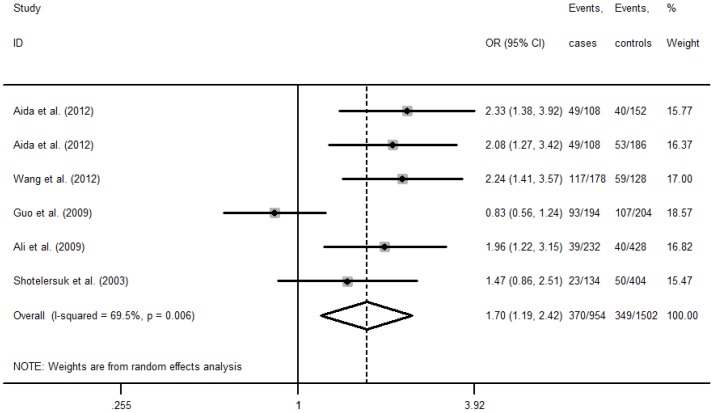
Forest plot of association between MTHFR A1298C polymorphism and NSCL/P risk in mothers (T vs. C).

The association between MTHFR C677T and NSCL/P risk was shown in Table 2. Significant heterogeneity between studies was found in the majority of the genetic comparisons, except for allele comparison (T vs. C: I^2^ = 42.3%), homozygote comparison (TT vs. CC: I^2^ = 28.3%) and recessive comparison (TT vs. CC/CT: I^2^ = 17.4%) in Asian children, and recessive comparison (TT vs. CC/CT: I^2^ = 49.8%) in Asian mothers. Overall, the variant T allele of MTHFR C677T increased risk of NSCL/P, when compared with the wild-type C allele. The OR (95%CI) was 1.41(1.23–1.61, P<0.001, Figure 2) in Asian children, and 1.70(1.19–2.42, P = 0.003, Figure 3) in Asian mothers, respectively. In the subgroup analyses of geographical location, the similar associations were also observed in Eastern Asian children, Western/Central Asian children and mothers, but not in Eastern Asian mothers.

**Table 2:** Overall and subgroup results of the association between MTHFR C677T polymorphism and NSCL/P.

Study(n)	T vs. C		TT vs. CC		CT vs. CC		CT/TT vs. CC		TT vs. CC/CT	
	OR(95%CI)	*P*	OR(95%CI)	*P*	OR(95%CI)	*P*	OR(95%CI)	*P*	OR(95%CI)	*P*
**Overall**										
Children(n = 8)	**1.41(1.23–1.61)**	**<0.001**	**1.85(1.30–2.63)**	**0.001**	**1.61(1.21–2.14)**	**0.001**	**1.61(1.22–2.11)**	**0.001**	1.36(1.00–1.86)	0.052
Mothers(n = 6)	**1.70(1.19–2.42)**	**0.003**	**2.58(1.09–6.09)**	**0.031**	**1.66(1.08–2.55)**	**0.021**	**1.83(1.17–2.88)**	**0.008**	**1.95(1.32–2.86)**	**0.001**
**Eastern Asia**										
Children(n = 4)	1.31(0.95–1.80)	0.099	**1.79(1.16–2.76)**	0.009	1.68(0.88–3.22)	0.117	1.66(0.89–3.10)	0.111	1.26(0.87–1.83)	0.217
Mothers(n = 3)	1.39(0.75–2.54)	0.293	2.19(0.46–10.44)	0.326	1.09(0.72–1.65)	0.688	1.28(0.64–2.59)	0.487	2.06(0.64–6.61)	0.224
**Western/Central Asia**										
Children(n = 4)	**1.50(1.25–1.81)**	**<0.001**	**1.96(1.08–3.55)**	0.026	**1.56(1.25–1.95)**	**<0.001**	**1.61(1.30–1.99)**	**<0.001**	1.44(0.53–3.95)	0.477
Mothers(n = 3)	**2.11(1.58–2.81)**	**<0.001**	**3.66(1.78–7.52)**	**<0.001**	**2.40(1.64–3.50)**	**<0.001**	**2.53(1.75–3.64)**	**<0.001**	**2.38(1.22–4.66)**	**<0.001**

For MTHFR A1298C polymorphism, the main results were shown in Table 3. Sound homogeneity between studies was seen, except for allele comparison (C vs. A: I^2^ = 71.0%), heterozygote comparison (AC vs. AA: I^2^ = 76.0%) and dominant comparison (AC/CC vs. AA: I^2^ = 74.8%) in Asian children. However, no significant associations between MTHFR A1298C polymorphism and NSCL/P were found under any of genetic model, even in the subgroup analyses by geographical location, neither in Asian children nor in Asian mothers.

**Table 3:** Overall and subgroup results of the association between MTHFR A1298C polymorphism and NSCL/P.

Study(n)	T vs. C		TT vs. CC		CT vs. CC		CT/TT vs. CC		TT vs. CC/CT	
	OR(95%CI)	*P*	OR(95%CI)	*P*	OR(95%CI)	*P*	OR(95%CI)	*P*	OR(95%CI)	*P*
**Overall**										
Children(n = 6)	1.03(0.79–1.35)	0.819	1.08(0.78–1.48)	0.652	1.01(0.68–1.48)	0.982	1.03(0.71–1.48)	0.881	1.09(0.80–1.48)	0.578
Mothers(n = 3)	0.94(0.73–1.20)	0.593	0.78(0.40–1.50)	0.452	0.98(0.71–1.35)	0.905	0.95(0.70–1.30)	0.751	0.79(0.41–1.50)	0.467
**Eastern Asia**										
Children(n = 3)	1.05(0.61–1.82)	0.855	0.97(0.47–1.99)	0.930	1.16(0.44–3.06)	0.767	1.13(0.49–2.61)	0.768	0.96(0.48–1.93)	0.907
Mothers(n = 1)										0.224
**Western/Central Asia**										
Children(n = 3)	1.04(0.73–1.49)	0.819	1.23(0.53–2.85)	0.626	0.94(0.76–1.16)	0.551	0.98 (0.66–1.44)	0.901	1.23(0.60–2.53)	0.568
Mothers(n = 2)	0.83(0.62–1.12)	0.218	0.81(0.20–3.27)	0.767	0.81(0.54–1.20)	0.283	0.79(0.54–1.15)	0.218	0.88(0.24–3.17)	0.839

### Sensitivity analysis and publication bias

**Figure 4:**
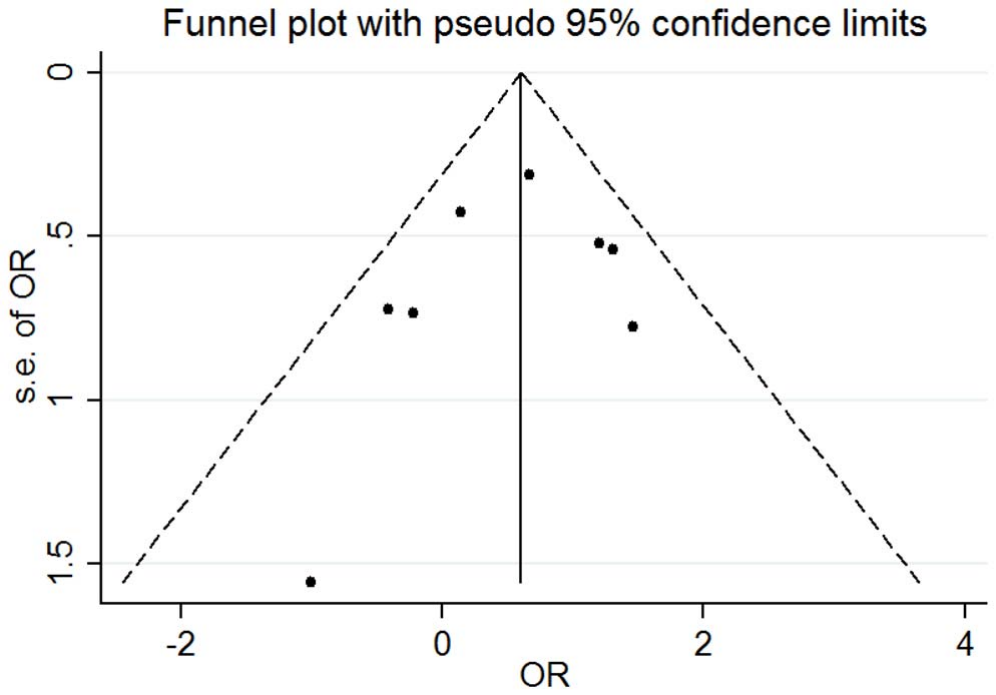
Funnel plot analysis to detect publication bias.

Elimination of each study made no qualitative difference on the pooled OR values, which indicated that the final results of the meta-analysis were stable. The publication bias of the included studies was assessed by the Funnel plot and Egger's test. The shapes of Funnel plot in each genotype comparison indicated no obvious asymmetry (Figure 4). And Egger's test provided statistical evidence for the funnel plot symmetry. No significant publication bias was found in the studies.

## Discussion

In this meta-analysis, we investigated the association between MTHFR C677T and A1298C polymorphisms and NSCL/P, with a total of nine case-control studies included. The pooled results indicated that there was an obvious association between MTHFR C677T polymorphism and NSCL/P risk in Asian children under all models: allele contrast (T vs. C), homozygote (TT vs. CC), heterozygote (CT vs. CC) and dominant (TT/CT vs. CC) models, except for recessive (TT vs. CT+CC) model. Meanwhile, the variant T allele of Asian mothers was significantly associated with a 1.70-fold increased risk of having a NSCL/P offspring. Subgroup analyses by geographical location further identified this association in Eastern Asian children, Western/Central Asian children and mothers, but not in Eastern Asian mothers. Regarding MTHFR A1298C polymorphism, the pooled results revealed that A1298C was not related to children's or mothers' NSCL/P susceptibility under any of the genetic model, even in the subgroup analyses by geographical location. The above results suggested that MTHFR C677T polymorphism was a risk factor in the development of NSCL/P in Asian populations.

As far as we know, there has been four published meta-analyses regarding MTHFR polymorphisms and NSCL/P risk. According to Verkleij-Hagoort et al.(2007) [19] and Johnson and Little (2008) [20], no significant associations between MTHFR C677T and A1298C polymorphisms and NSCL/P were acquired. The pooled results of Luo Y et al. (2012) [17] indicated that maternal MTHFR 677TT genotype was related to increased risk of having a NSCL/P offspring. The more recently meta-analysis conducted by Pan Y et al. (2012) [18] showed that MTHFR C677T polymorphism contributed to elevated risk of NSCL/P among Asians. Compared with the previous meta-analyses, we updated this meta-analysis by adding newly published studies, which were not included in the previous meta-analyses. Finally, we achieved consistent conclusions with Luo Y et al. (2012) and Pan Y et al. (2012).

Some limitations of this meta-analysis should be acknowledged. Firstly, the sources of control among the studies were different from each other. Some studies were population-based studies, and others were hospital-based studies. Secondly, our results were based on unadjusted OR values that lack the original data from the eligible studies, which could lead to relatively weak power to estimate the real relationship. Thirdly, our analyses were based on single-factor estimates, which overlooked the interactions of gene-gene and gene-environment in the development of NSCL/P. Finally, the sample size was relatively small to investigate the association between MTHFR C677T and A1298C polymorphisms and NSCL/P risk.

In conclusion, our meta-analysis suggested that MTHFR C677T polymorphism was related with an increased NSCL/P risk in Asian populations. In the future, large sample studies should be warranted to investigate the association between MTHFR C677T and A1298C polymorphisms and NSCL/P, and to examine the potential gene-gene and gene-environment interactions.

## Supporting Information

Checklist S1 (DOC)
